# Sectional Medicine

**Published:** 1849-01

**Authors:** 


### ARTICLE V.

### SECTIONAL MEDICINE.

The old controversy, in reference to the advantages of studying medicine in the region of country in which the student expects to practice, has been revived by the use of the following language in the last circular of Jefferson Medical College, Philadelphia, viz:—

The idea that a Student of Medicine must be taught his profession in the very locality in which he is destined to practice it, is now generally, as it ought to be universally, abandoned. It must be admitted that the great principles of the science are the same everywhere, and that the Student ought, for his own sake, to seek for information wherever it can be best and most readily obtained.

To this our cotemporaries of the South. Med. and Surg. Jour., and of the New Orleans Med. and Surg. Jour., take exceptions, and we think with much show of reason.

The Medical Examiner espouses the side of the question taken by the circular quoted, and is assisted by the correspondence of B. Rush Mitchell, M. D., who refers to the success of the surgeons of the navy in treating yellow fever during the service on the Gulf of Mexico during the Mexican war, to illustrate the position that they can teach students in eastern schools to be successful practitioners in the South.

The ground that the general principles of our science are of universal application so much harped upon by those who oppose what they term "sectional medicine," is undoubtedly correct; but, in our humble opinion, does not cover the case under consideration. No one, we trust, will contend that diseases are the same in all places, and under all circumstances.

Here, if we understand the position correctly, it is contended that in teaching the application of those principles in practice, some experience, some actual observation of cases is necessary to qualify the teacher to teach, and the student to practice. And further, the more extensive the observation of any given class of cases, the bettei the qualifications of both in reference to them.

In this, we do but assert what all admit as being true in reference to the whole range of diseases; and if so, how can it be otherwise than true in reference to a class of them. Therefore, other things being equal, the western teacher is better qualified to teach the management of all those forms of disease prevalent in this region; and so of the East and South.

What qualifies Ricord to teach the pathology and treatment of venereal diseases, but his great experience in such cases, and his extensive means of illustration in his ward. Why are his students qualified to treat such cases? Because they were taught by one who was thoroughly familiar with them, and because they have seen for themselves. If true, in reference to this, certainly it is so in reference to the fevers of the West and South.

The recommendation of the American Medical Association, that students should have clinical instruction to qualify them for practice, places that body on the side of this question that we advocate.

What is the object of clinical teaching? It can only be to make the student familiar with the symptoms, forms, and characters of diseases, and the effects of remedies when applied in their treatment. And it is necessary that each disease be studied for itself. There are no general principles in pathology that lead us to understand one disease from having studied another. No one would understand a fever from having studied cholera. To learn to recognize and treat typhus fever, it must be studied and observed for itself; and so with each other fever. Then sectional or not, we see no reason why it is improper to say that to learn to understand and manage remittent, intermittent, congestive, or yellow fever, the student must be taught of one who is familiar with it; and to receive clinical instruction, in reference to either, he must go where it prevails.

Then to advocate the importance of clinical teaching, advocates the advantages to the student of studying diseases "in the very locality in which he is destined to practice."

We believe in the sentiment of Macaulay, as quoted by the New Orleans Med. and Surg. Jour., and expressed in the following:—

> But what should we think of a physician who should now tell us that he deduced his treatment of yellow fever from the general theory of pathology? Surely we should ask him, whether, in constructing his theory of pathology, he had, or had not, taken into the account the facts which had been ascertained respecting the yellow fever? If he had, then it would be more correct to say, that he had arrived at the principles of pathology partly by his experience of cases of yellow fever, than he had deduced his treatment of yellow fever from the principles of pathology. If he had not, he should not prescribe for us. If we had the yellow fever, we should prefer a man who had never treated any cases but cases of yellow fever, to a man who had walked the hospitals of London and Paris for years, but who knew nothing of our particular disease.

In regard to the general abandonment of the position, we think it a mistake; and, although one of the editors of the St. Louis Medical Journal thinks medical schools should base their claims for patronage on higher grounds, we are quite sure that most of our western practitioners will say that in addition to the ability of teachers, and the means of illustrating the elementary branches, familiarity with those diseases the student must meet in practice, and the means of giving clinical instruction on them, will constitute the very highest possible ground for preferring one school over another. E.

